# Editorial: Sexual dimorphism in biomedical research and its therapeutic implications

**DOI:** 10.3389/fendo.2022.1009712

**Published:** 2022-08-19

**Authors:** Jan Kroon, Shokoufeh Mahmoodzadeh, Christine Helsen

**Affiliations:** ^1^ Division of Endocrinology, Department of Medicine, Leiden University Medical Center, Leiden, Netherlands; ^2^ Division of Molecular Biology of Peptide Hormones in Cardiovascular System, Max-Delbrueck-Center for Molecular Medicine in the Helmholtz Association, Berlin, Germany; ^3^ Department of Cellular and Molecular Medicine, Faculty of Medicine, KU Leuven, Leuven, Belgium

**Keywords:** sexual dimorphism, glucocorticoids, personalized therapy, steroid hormones, sex chromosomes, nuclear receptors

Substantial sex differences are known to exist in various diseases and conditions with sex affecting the incidence, the progression and the biology of the disease (1–3). Focusing on these sex differences and their underlying mechanisms is therefore a crucial aspect in biomedical research and will aid in further advancing therapeutic options for men and women. Multiple factors can contribute to sexual dimorphism such as the differences in organ and tissue development between male and female, the difference in genetic landscape due to sex chromosomes, but also the difference in the availability of sex hormones and their receptors. While the androgen receptor (AR) typically responds to the presence of testosterone and the estrogen receptor (ER) is activated by estrogens, a new complexity of cross-talk between steroid receptors has been suggested to modulate steroid receptor signaling and could be involved in sexual dimorphism. In this Research Topic ‘Sexual dimorphism and steroid hormone crosstalk’, we have gathered a collection of original research articles and reviews that investigate sexual dimorphism in a range of diseases and processes including malaria, glucocorticoid-induced muscle atrophy, brown adipose tissue dysfunction and high-fat diet-induced metabolic disease.

To further define potential mechanisms underlying sex dimorphism, it is important to link the clinical observations to biomedical findings, more precisely changes in the protein or mRNA level or the DNA binding pattern of the protein of interest. In the article by McLeod et al., they investigated the role of AR in motor neuron vulnerability in the SOD1^G93A^ mouse model of amyotrophic lateral sclerosis. By mapping AR expression throughout the central nervous system of male mice, a reduction of nuclear AR in the lumbar spinal motor neurons was observed to be present already at a pre-symptomatic age.

In referring to sexual dimorphism in diet-induced obesity, Iena et al. describe that females remain less susceptible to a high-fat diet compared to male mice due to a sex-specific transcriptional regulation of the aquaporin 7 channel that is involved in glycerol transport in white adipose tissue. This increases efflux of glycerol from adipose tissue and as a result leads to a less severe accumulation of triglycerides in female white adipose tissue depots. Male AQP7 expression remained unaffected in response to high-fat diet.

It is important to realize that receptors for steroid hormones are expressed beyond their classically recognized (reproductive) target tissues, and that also other peripheral and central tissues have relevant expression levels of e.g. the androgen and estrogen receptor (and are therefore potentially responsive to sex hormones). For example, brown adipose tissue, a key metabolic and thermogenic tissue, expresses both the AR and the ER-α. It may therefore come to no surprise that sex differences were described in brown adipose tissue biology including volume, distribution in the body, cellular composition and thermogenic capacity of the tissue, as reviewed in Kaikaew et al..

As a case in point, glucocorticoids represent a class of widely used drugs in the clinic and are mainly applied for their strong anti-inflammatory actions. Sex differences in the potency of the synthetic glucocorticoids were found in a model for collagen-induced arthritis, in which dexamethasone prevented paw edema with an almost 10-fold lower potency in female as compared to male rats (4). Similarly, the sensitivity to betamethasone-induced muscle atrophy was lower in female mice as compared to male mice (Li et al.). It is important to note that different glucocorticoids can have different effects, in particular when comparing endogenous glucocorticoids (such as cortisol and corticosterone) that also bind the mineralocorticoid receptor in addition to the glucocorticoid receptor; and synthetic glucocorticoids (like dexamethasone and betamethasone) which are typically more specific for the glucocorticoid receptor (with little mineralocorticoid activity at clinically relevant doses). Given a driving role of deregulated glucocorticoid signaling in many diseases, also glucocorticoid receptor antagonists have utility, but also for this class of drugs sex differences (in therapeutic efficacy) were found (e.g. on the capacity to antagonize dexamethasone-induced gene upregulation) (5).

Gender disparities are also observed for urothelial bladder cancer where men have a higher incidence of the disease but women show highest mortality rates. The latter could be due to a higher prevalence of the basal-squamous subtype in women which is associated with a poor prognosis. While the underlying mechanism of this sexual dimorphism remains uncovered, Goto et al. provide an extensive overview of the potential role of estrogen receptor-alpha and -beta in urothelial bladder cancer in their latest review.

The need to include sex as a variable in clinical and laboratory studies is also demonstrated by the study of Cervantes-Candelas et al., showing that the innate and adaptive immune responses to malaria are influenced by sex hormones. Understanding the mechanisms underlying sexual dimorphism in the immune response is the first step toward individualized therapy and vaccines that take into account the sex of the individual, and this could result in more effective or less toxic formulations.

## Author contributions

JK and CH wrote the editorial. SM provided feedback on the editorial. All authors read and approved the final version of the editorial.

## Acknowledgments

We acknowledge all contributing authors in this Research Topic.

## Conflict of interest

JK is sponsored by Corcept Therapeutics.

The remaining authors declare that the research was conducted in the absence of any commercial or financial relationships that could be construed as a potential conflict of interest.

## Publisher’s note

All claims expressed in this article are solely those of the authors and do not necessarily represent those of their affiliated organizations, or those of the publisher, the editors and the reviewers. Any product that may be evaluated in this article, or claim that may be made by its manufacturer, is not guaranteed or endorsed by the publisher.

## References

[1] Reue K Wiese CB. Illuminating the mechanisms underlying sex differences in cardiovascular disease. Circ Res (2022) 130:1747–62. doi: 10.1161/CIRCRESAHA.122.320259 PMC9202078 35679362

[2] Chlamydas S Markouli M Strepkos D Piperi C. Epigenetic mechanisms regulate sex-specific bias in disease manifestations. J Mol Med (Berl) (2022) 100:1111–23. doi: 10.1007/s00109-022-02227-x. 20220629 PMC9244100 35764820

[3] Tokatli MR Sisti LG Marziali E Nachira L Rossi MF Amantea C. Hormones and sex-specific medicine in human physiopathology. Biomolecules (2022) 12:20220307. doi: 10.3390/biom12030413 PMC8946266 35327605

[4] Song D DuBois DC Almon RR Jusko WJ. Modeling sex differences in anti-inflammatory effects of dexamethasone in arthritic rats. Pharm Res (2018) 35:203. doi: 10.1007/s11095-018-2483-5. 20180906 30191329 PMC7197054

[5] Kroon J Viho EMG Gentenaar M Koorneef LL van Kooten C Rensen PCN. The development of novel glucocorticoid receptor antagonists: From rational chemical design to therapeutic efficacy in metabolic disease models. Pharmacol Res (2021) 168:105588. doi: 10.1016/j.phrs.2021.105588. 20210331 33798733

